# High Genetic Connectivity and Weak Structure Shape Australian White Ibis Metapopulation Dynamics Across an Urban Coastal Landscape

**DOI:** 10.1002/ece3.74247

**Published:** 2026-08-28

**Authors:** Dominique A. Potvin, Angela G. Webb, Tatiana Wendy Fonseca, Jessica Gorring, Caitlin S. Willis, Erin K. Wills, Ben L. Gilby

**Affiliations:** ^1^ School of Science, Technology and Engineering University of the Sunshine Coast Petrie Queensland Australia; ^2^ Centre for BioInnovation University of the Sunshine Coast Sippy Downs Queensland Australia; ^3^ School of the Environment University of Queensland St Lucia Queensland Australia; ^4^ City of Moreton Bay Caboolture Queensland Australia

**Keywords:** dispersal, gene flow, population genetics, sex ratios, *Threskiornis*, urban ecology

## Abstract

Urbanisation reshapes habitat structure, resource availability, and movement pathways for many wildlife species, often creating novel selective pressures and management challenges. The Australian white ibis ( *Threskiornis molucca* ) has rapidly expanded from its historic range into eastern Australian cities, causing some conflicts with people, yet the genetic and demographic processes underpinning this shift remain poorly understood. We used SNP‐based genomic data from 184 individuals sampled across 15 colonies in the Moreton Bay City region of Southeast Queensland to investigate population structure, genetic diversity, inbreeding, dispersal, and landscape resistance. Genetic diversity was moderate across colonies (*H*_e_ 0.134–0.166; *H*_o_ 0.084–0.145), and pairwise *F*_ST_ values were uniformly low (< 0.14), consistent with widespread gene flow, though two colonies exhibited elevated inbreeding (*F*_IS_ ≤ 0.28), suggesting local dispersal constraints. Kinship analyses confirmed both within‐ and cross‐colony sibling and parental relatedness, providing direct evidence of recent interbreeding and movement. We found a balanced sex ratio and no evidence of sex‐biased dispersal. Landscape resistance models revealed that genetic differentiation was only weakly associated with habitat variables, a striking contrast to many urban adaptation studies where anthropogenic features typically fragment populations. This suggests that urban development may paradoxically facilitate connectivity for this species by creating a network of resource‐rich foraging and breeding colonies that promote movement. Together, these results demonstrate that ibis in this region form a single, interconnected population with high gene flow and demographic resilience, rather than a classic metapopulation. Management seeking to minimise human‐ibis conflict therefore requires coordinated, landscape‐scale strategies addressing resource availability and inter‐site movement, rather than colony‐specific approaches alone.

## Graphical abstract

Australian white ibis ( *Threskiornis molucca* ) is now ubiquitous in urban landscapes in Southeast Queensland. However, whether individual colonies might represent genetically congruous or disparate populations, or whether genetic connectivity might be driven by habitat or landscape features is unknown. We find evidence that across the urban matrix, ibis colonies are highly genetically connected, with habitat features such as wetlands potentially serving to semi‐isolate some populations.

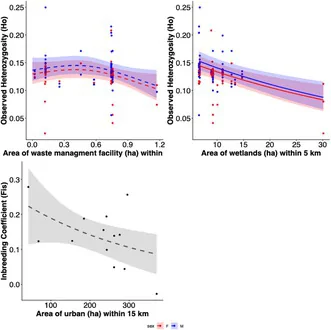

## 1 Introduction

Urbanisation is one of the most pervasive forms of landscape change worldwide, reshaping habitat availability, movement pathways, dispersal opportunities, and ultimately gene flow for many wildlife species (Elmqvist et al. 2013; McCleery et al. 2014). These shifts can either restrict or enhance connectivity depending on species' behavioural flexibility and the configuration of the urban matrix. Within this broader context, the eastern Australian coastline has undergone dramatic, large‐scale urbanisation over the last 40 years and continues to experience high rates of development (Evans 2016; Garden et al. 2006; Lee et al. 2006). While urbanisation can be detrimental to many native species (Garden et al. 2006; Mosman et al. 2024; O'Donnell and delBarco‐Trillo 2020; Taylor‐Brown et al. 2019), urban centres can also act as refuges, providing a rich abundance of habitat, food and water resources and protection from predation (Boer‐Cueva et al. 2025; Martin and Major 2010; Murray et al. 2013). In this time, the Australian white ibis ( *Threskiornis molucca* ; hereafter ‘ibis’) has adapted to anthropogenic environments, forming large colonies throughout coastal cities, likely following the degradation of inland wetlands (Corben 2008; Martin et al. 2010; Martin et al. 2011; Smith et al. 2013; Smith and Munro 2011). This shift in habitat use may reflect altered source‐sink dynamics, where certain colonies act as demographic sources and others as sinks.

Now widespread and ubiquitous across most major Australian east coast cities, ibis commonly aggregate, roost and nest in landfills, parks, constructed wetlands, roadside vegetation, and airports (Corben and Munro 2006; Corben 2008; Willis et al. 2024), forming colonies with up to hundreds of nests and thousands of individuals (Corben and Munro 2006; Martin et al. 2007; Shaw 1999). As a result, ibis in urban areas pose elevated risks of vehicle and aircraft collisions (Davis et al. 2021; Shaw 1999), and raise concerns about disease dynamics and resource overexploitation in densely occupied habitats (Epstein et al. 2006; Maute et al. 2019; Davis et al. 2021; Underwood and Bunce 2004). Despite their increasing abundance and potentially expanding conflicts with humans across landscapes, knowledge gaps remain regarding the behavioural and ecological factors that support ibis population persistence and expansion within urban environments.

Ibis are habitat generalists with high levels of behavioural plasticity, particularly apparent in their foraging behaviours (Corben 2008; Martin et al. 2011; Murray and Shaw 2009). Urban ibis use both visual and non‐visual (e.g., probing into concealed substrates) techniques to derive food from various natural and anthropogenic sources and in different land use types (Marchant and Higgins 1990; Murray and Shaw 2009). Waste management facilities and landfills serve as key foraging sites for urban ibis, offering abundant and stable food resources and often supporting the highest foraging densities (Martin et al. 2010; Willis et al. 2024). Despite a potential trade‐off for dietary quality, the provision of stable and abundant resources in urban environments allow urban ibis populations to exhibit higher reproductive output throughout the year, along with greater site fidelity, and shorter foraging distances compared to non‐urban populations (Martin et al. 2012; Martin et al. 2011; Smith et al. 2013). This suggests that the quantity and predictability of food resources are more important than food or foraging site quality in driving local population growth and success, a pattern observed in other human commensal avian species (Gil and Brumm 2014), and that such anthropogenic drivers of home ranges and distributions might therefore drive different genetic structure between urban and non‐urban populations.

Landscape features influencing resource availability may also affect patterns of dispersal and genetic diversity. Wetlands represent the natural breeding and foraging habitat of ibis, whereas waste‐management facilities provide predictable anthropogenic food resources that can attract large numbers of birds (Martin et al. 2010; Willis et al. 2024). Urban land cover may similarly alter movement pathways by influencing the distribution and accessibility of resources across the landscape. Consequently, variation in these landscape features may affect both connectivity among colonies and the degree of genetic mixing occurring within colonies.

Physical barriers, resource availability and urban management practices can strongly influence genetic connectivity between urban and non‐urban ibis populations (Davis et al. 2021). Increasing landscape heterogeneity caused by expanding urbanisation can alter source‐sink metapopulation dynamics, whereby some colonies produce excess individuals that disperse to other sites, while others persist only through immigration (Balogh et al. 2011; Kanda et al. 2009). Although urban areas can enhance resource availability and support higher reproductive output, non‐urban colonies may experience reduced effective population sizes and associated genetic risks, including increased inbreeding in isolated sites or outbreeding depression in highly mixed colonies (Lowe and Allendorf 2010). At the same time, the long‐term viability of urban colonies may be constrained by reduced evolutionary capacity (Frankham 2005; Lowe and Allendorf 2010). Colony dynamics and dispersal may also be influenced by sex ratios, particularly if urban environments promote sex‐biased recruitment or survival (Li and Kokko 2019). Identifying levels of inbreeding or outbreeding, colony sex ratios and genetic connectivity across sites in the overall landscape can help in assessing long‐term population viability and understanding broader metapopulation consequences under increasing habitat fragmentation (Battin 2004; Donovan et al. 1995; Edmands 2007; Hale and Swearer 2016).

Genetic information can be used to measure connectivity between individuals and subpopulations or colonies, providing insights into metapopulation dynamics and contributions of habitat to overall population stability (De Young and Honeycutt 2005). It is also valuable for informing management strategies, particularly for species with high dispersal capacity or migratory behaviours. For example, for species with continuous distributions, relying solely on habitat‐ or location‐based definitions of colony boundaries can lead to inaccurate estimates of demographic structure or distribution, whereas genetic data can more reliably delineate biologically meaningful units (De Young and Honeycutt 2005). Thus, colony and population genetic datasets are powerful tools for ecology and wildlife management, especially when integrated with behavioural, demographic, and spatial information.

Here, we use SNP‐based genomic data to evaluate how urbanisation shapes ibis population processes, focusing on three core hypotheses. First (H1), because urban ibis routinely exploit predictable resources and move among sites, we expected weak population structure and high connectivity among colonies. Second (H2), given frequent movements and the species' capacity for regional mixing, we predicted high genetic diversity and low inbreeding across the landscape, as well as demographic indicators consistent with widespread dispersal (e.g., cross‐colony close kin, broadly similar effective population sizes, and no strong sex‐biased movement). Third (H3), given the mobility of ibis in human‐modified environments, we anticipated weak landscape resistance, with landscape composition explaining little additional variation in genetic differentiation beyond geographic distance. However, we expected that variation in wetlands, urban land cover, and waste‐management facilities could influence local movement behaviour and therefore contribute to variation in genetic diversity and inbreeding among colonies. Together, these hypotheses build on prior evidence of extensive movement among urban and non‐urban ibis populations and provide a framework for integrating genomic, spatial, and demographic indicators to assess colony connectivity and long‐term viability in this system. Given the extensive dispersal previously reported for ibis, an alternative possibility is that colonies may function as components of a single highly connected regional population rather than as genetically distinct metapopulation units.

## 2 Methods

### 2.1 Study Species and Sampling

The Australian white ibis ( *Threskiornis molucca* ), a large waterbird of the order Pelecaniformes, is native to freshwater wetlands, estuaries, and grasslands but has adapted to urban environments such as parks and landfills (Marchant and Higgins 1990; Martin et al. 2010; Smith et al. 2013). Ibis nest colonially in vegetation near water bodies (Carrick 1962; Smith et al. 2013; Willis et al. 2024) and exhibit year‐round breeding in urban areas, contrasting with seasonal breeding in natural habitats (Kingsford and Johnson 1998; Martin et al. 2007).

**FIGURE 1:**
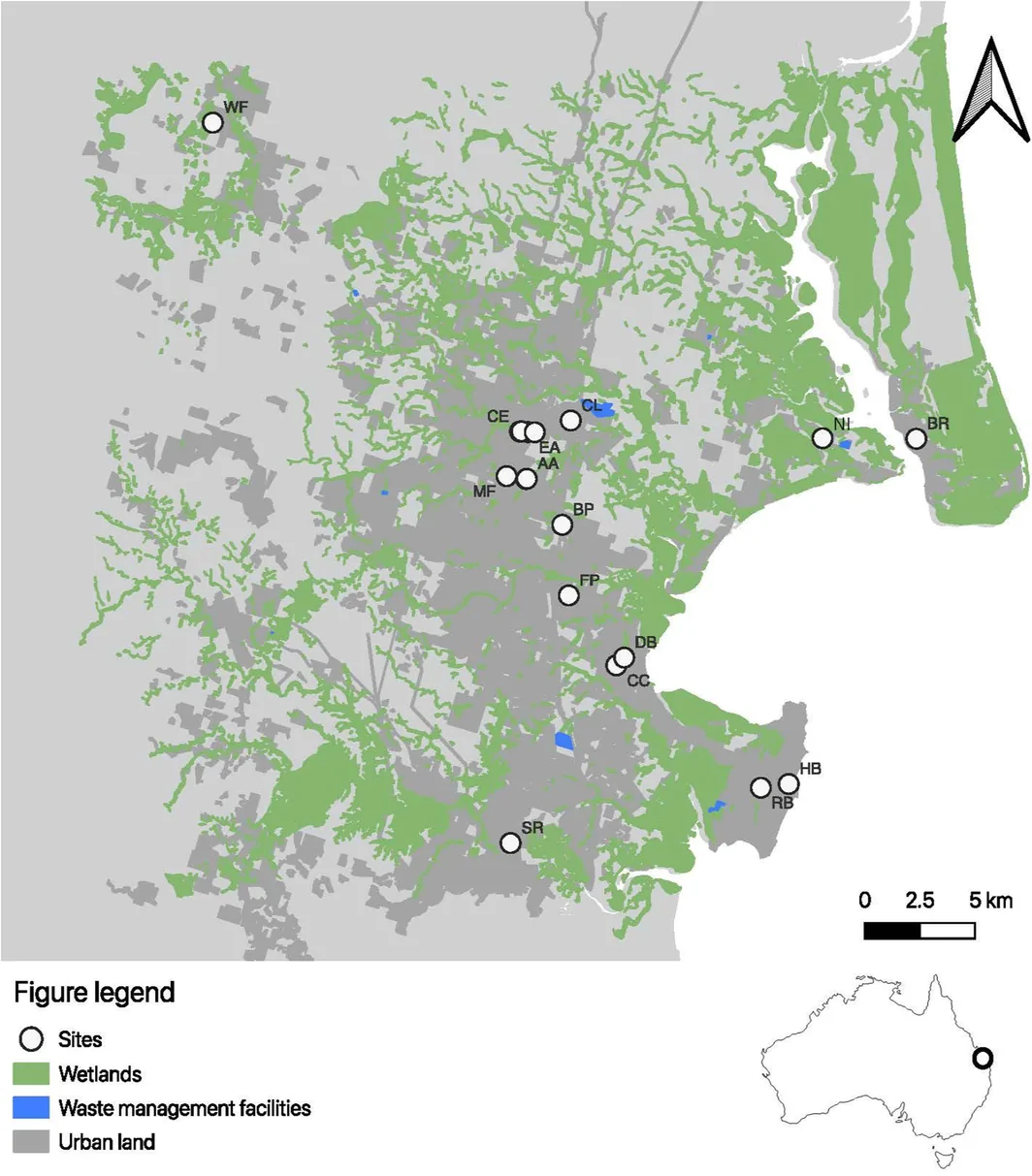
Map of ibis survey sites in Southeast Queensland, Australia, with land use types indicated. Site codes as follows: AA = Arthur Allan; BP = BP Caboolture; BR = Bribie; CE = Centenary; CL = Central Lakes; CC = Clough Ct; DB = DBay SES; EA = Esme Ave; FP = Fodora Place; HB = Humpybong; MF = Morayfield; NI = Ningi; RB = Redcliffe Bot Gar; SR = Sweeney Res; WF = Woodford.

Between February 2022 and November 2024, we sampled 184 ibis across 15 sites in the Moreton Bay Region, Queensland, Australia (Figure 1) representing diverse land‐use types. All sampled colonies occurred within the Moreton Bay coastal region. Additional colonies occur within this region; however, the colonies included in this study were selected based on accessibility, active occupancy, and to capture variation in surrounding landscape composition. Sampled colonies were distributed across a range of urban and peri‐urban settings that differed in the relative abundance of wetlands, urban land cover, and waste‐management facilities.

Individuals were captured using hand nets, cannon nets, or leg nooses, depending on location. Each bird was processed following Australian Bird and Bat Banding Scheme (ABBBS) protocols: morphological measurements were recorded, ~20 μL of blood was drawn from the brachial vein (stored in 95% ethanol), and an individually identifiable stainless‐steel leg band was fitted. Feathers were non‐invasively collected from eight sites to supplement genetic data.

Sample sizes differed among colonies due to variation in colony size, accessibility, and capture success. Several colonies were represented by only two or three individuals. These colonies were retained to maximise spatial coverage across the study region, although colony‐level estimates derived from these small samples should be interpreted cautiously because of increased sampling uncertainty. Throughout this manuscript, we use *site* to refer to a geographic sampling location, *colony* to refer to the breeding aggregation occupying that location, and *population* to refer to the broader regional genetic population encompassing all sampled colonies.

### 2.2 DNA Extraction, Sequencing, and PCR Sex Determination

DNA was extracted from blood and feather samples using a Qiagen DNeasy Blood & Tissue Kit following the manufacturer's protocols, and DNA integrity was assessed via 1.5% agarose gel electrophoresis. Genotyping was performed by Diversity Arrays Technology using their proprietary DArTseq platform, which applies genome complexity reduction with *PstI* and *SphI* restriction enzymes (Cruz et al. 2013), producing a high‐density SNP dataset suitable for population analyses.

We sexed a subset of 120 individuals, using CHD1F/CHD1R primers (J. C.‐I. Lee et al. 2010) to amplify the sex‐linked CHD1‐Z/W genes. PCR reactions (15–25 μL) consisted of ~20 ng template DNA, 1× PCR buffer, 2.0–2.5 mM MgCl_2_, 0.2 mM dNTPs, 0.4 μM of each primer, and 0.5 U Taq polymerase. Thermocycling conditions followed Lee et al. (2010): initial denaturation at 94°C for 2 min; 35 cycles of 94°C for 30 s, 50°C–52°C for 45 s, and 72°C for 45 s; final extension at 72°C for 5 min. Amplicons were visualised on 1% agarose gels: males displayed a single ~500 bp band (CHD‐Z), whereas females showed two bands (~500 and ~700 bp; CHD‐Z and CHD‐W).

### 2.3 Bioinformatic and Population Genetic Analyses

We analysed the SNP data received using the ‘dartR’ package (Gruber et al. 2018; Mijangos et al. 2022) in R v4.4.1 (R Core Team 2024). Genotyping initially yielded 55,896 SNP loci across 184 individuals from 15 colonies. The raw dataset contained 17.18% missing data and an average locus call rate of 0.83 (median = 0.91). SNPs were filtered for reproducibility (> 90%), call rate (> 80%), read depth (5–50×), and tag length (20–69 bp), resulting in a final dataset of 27,649 loci (5.3% missing data).

To characterise population structure and connectivity (H1), we conducted principal component analysis (PCA) using ‘adegenet’ (Jombart 2008). Genetic differentiation among colonies was quantified using pairwise *F*_ST_ with 100 bootstrap replicates in ‘StAMPP’ (Pembleton et al. 2013). To test for isolation by distance (IBD), we performed Mantel tests comparing linearised *F*_ST_ (*F*_ST_/(1 − *F*_ST_)) and geographic distance matrices using the ‘vegan’ package (Oksanen et al. 2007).

To quantify genetic diversity and inbreeding (H2), we calculated observed heterozygosity (*H*_o_), expected heterozygosity (*H*_e_), and inbreeding coefficients (*F*_IS_) for each colony using ‘hierfstat’ (Goudet and Jombart 2013).

### 2.4 Sex‐Linked Marker Identification and Sex Assignment

For juveniles and other individuals not sexed using PCR, sex was inferred from SNP‐based sex‐linked markers, which allowed us to assign sex without additional laboratory assays. We compared allele frequencies between PCR‐confirmed adult males (*n* = 58) and females (*n* = 62) using ‘SNPrelate’ (Zheng et al. 2012), identifying 61 loci strongly differentiated between sexes (allele frequency difference > 0.5). These markers were used to assign sex probabilistically to 64 previously unsexed individuals by matching their multilocus genotypes to the male and female reference profiles. Reference profiles were constructed exclusively from PCR‐confirmed individuals sampled in the present study; individuals from unsampled colonies were not included in the sex‐assignment procedure. Because sex assignment was based on strongly differentiated sex‐linked loci, the approach is expected to be robust to variation among colonies within the study region.

Sex ratios were calculated for each colony and across all sampled individuals combined. To examine whether dispersal differed between sexes as part of the demographic assessment (H2), we repeated PCA and pairwise *F*_ST_ analyses separately for male and female subsets and compared patterns between them.

### 2.5 Kinship and Effective Population Size

To evaluate demographic processes associated with dispersal (H2), we estimated pairwise relatedness in *dartR* and identified close kin pairs (*R* > 0.18) within and between colonies. Pairwise relatedness was estimated in dartR to identify individuals that shared more alleles than expected under random mating. Relatedness coefficients provide an indication of recent shared ancestry, allowing the identification of potential parent–offspring and sibling relationships within and among colonies.

Effective population sizes (*N*_e_) were estimated for each colony using linkage disequilibrium methods. N_e_ was estimated using linkage disequilibrium (LD) methods, which infer the number of breeding individuals contributing genes to subsequent generations based on non‐random associations among loci. Smaller effective population sizes are expected to generate stronger LD because of increased genetic drift.

### 2.6 Landscape and Spatial Analyses

The influence of urban landscape features on genetic diversity and inbreeding was investigated using observed heterozygosity (*H*_o_) and inbreeding coefficients (*F*_IS_) as response variables (supporting H1). Spatial attributes expected to influence ibis movement, dispersal, and genetic mixing were compiled from landscape data (Willis et al. 2024). We measured the area of three landscape categories, being waste management facilities, urban land, and wetlands, within three radii (hereafter buffers) of 5 km, 10 km and 15 km surrounding each site (Willis et al. 2024). Further, we measured the distance (in meters) to the nearest waste management facility, urban land, wetland, and neighbouring colony.

### 2.7 Isolation by Resistance

We tested whether landscape resistance explained genetic differentiation beyond geographic distance alone (i.e., IBD, above) using colony‐level isolation by resistance (IBR). To do this, the pairwise F_ST_ matrix was aligned to colony GPS coordinates for all 15 sampled colonies. Resistance surfaces were generated across a bounding box buffered around the study extent at ~1 km resolution. The first surface (‘urban_proximity’) assigned resistance based on distance to the nearest colony centroid (cell values rescaled to 1–10); the second (‘landscape_variation’) was a random background surface (uniform 1–5; seed = 123). These resistance surfaces were intended as exploratory representations of spatial variation across the study landscape rather than mechanistic models of ibis movement. They were not directly parameterised using habitat variables known to influence ibis ecology, such as wetlands, urban land cover, or waste‐management facilities. For each surface, we computed least‐cost resistance distances between colonies using an 8‐neighbour transition matrix with geographic correction and we computed great‐circle geographic distances (km). We then compared pairwise genetic distances (*F*_ST_) to each resistance distance using Mantel tests (‘vegan’, permutations = 99), reporting Mantel *r* and permutation *p*‐values.

### 2.8 Spatial Patterns in Genetic Population Dynamics

To explore relationships between landscape composition, sex ratios, and genetic metrics, we fitted generalised additive mixed models (GAMMs) using *H*_o_ at the individual level and *F*_IS_ at the site level as response variables (supporting H2 and H3). GAMMs were fitted using thin plate regression splines in the *mgcv* package in R (Wood 2017). The *H*_o_ model included the fixed factors of sex and all spatial variables, as well as all two‐way interactions between sex and spatial variables and the random variable of ‘site’, being the location at which each individual was sampled from. The *F*_IS_ model included only the fixed spatial variables. Prior to analysis, two landscape variables, distance to wetland and distance to nearest colony, were log‐transformed to reduce the influence of outliers. All models were fit with the Gaussian error distribution. The best fit GAMM was identified using the full subsets approach in the *fssGAM* package (Fisher et al. 2018). Models with all possible combinations of four or fewer model terms were created, with the random ‘site’ variable force included for all subsets for the *H*_o_ model. The best fit model was the model with the lowest Akaike information criterion (AIC) value. The full subsets approach includes a step which prevents co‐varying variables from being in the same model, therefore eliminating any need for prior covariance checks.

### 2.9 Ethics

All procedures were approved by the University of the Sunshine Coast Animal Ethics Committee (ANA21189).

## 3 Results

Results are presented according to the three hypotheses outlined in the Introduction, progressing from analyses of population structure and connectivity (H1), to demographic and diversity patterns (H2), and finally landscape influences (H3).

### 3.1 Population Structure and Connectivity (H1)

After filtering, the dataset comprised 27,649 SNPs across 184 Australian white ibis, with ~5.3% missing data, providing sufficient genomic resolution for population‐genetic and landscape analyses.

**FIGURE 2:**
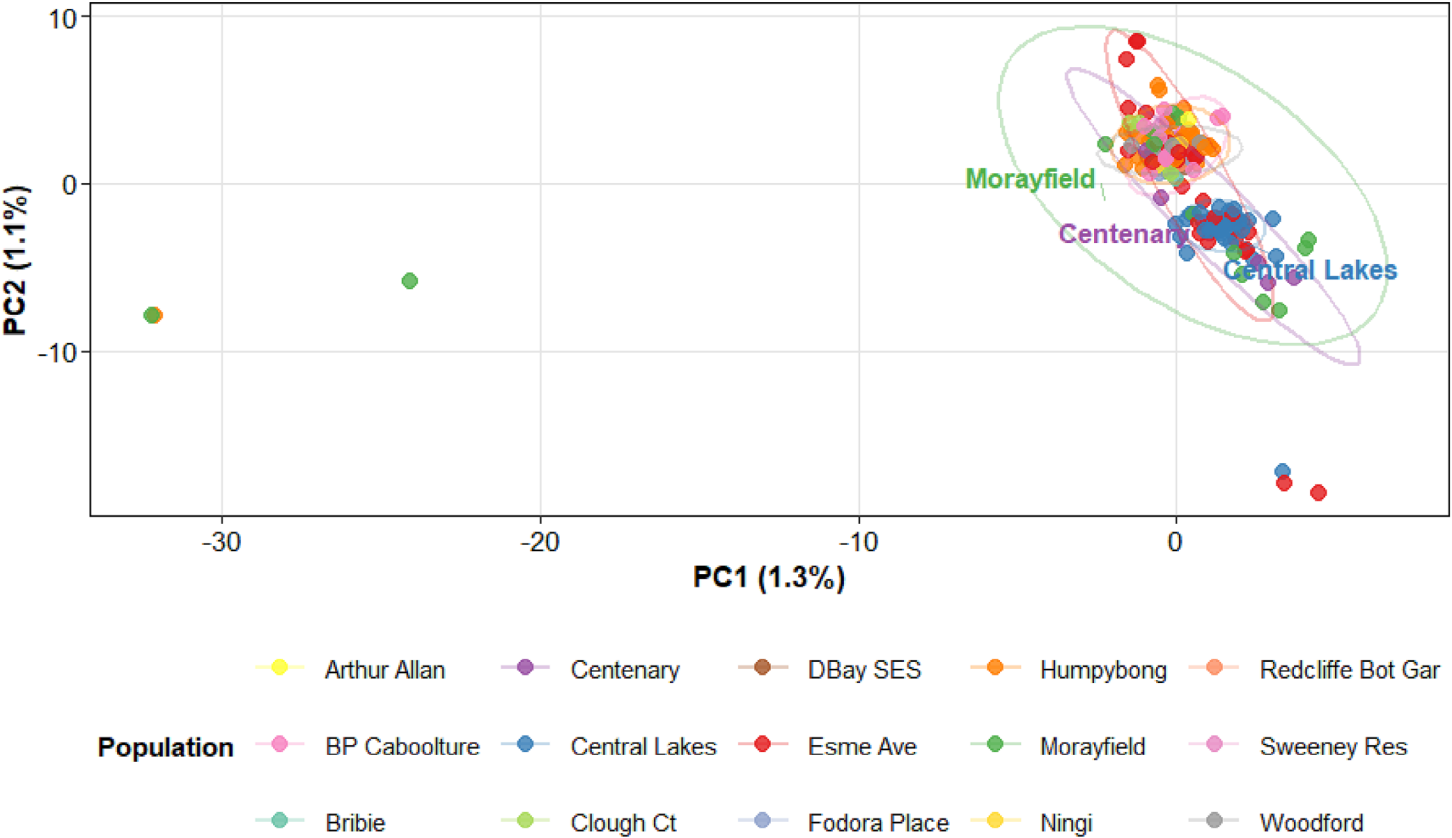
Principal Component Analysis (PCA) of Australian white ibis genotypes in the Moreton Bay region. The plot shows the first two principal components, explaining 1.3% and 1.1% of the total genetic variance, respectively. Each point represents an individual bird, coloured by population of origin. Ellipses represent 95% confidence intervals around each population centroid. Population labels of the largest sampled populations are positioned at group centroids.

Multivariate analyses revealed very weak population structure across the region. PCA explained minimal variation (PC1: 1.28%, PC2: 1.14%), with extensive overlap among colonies and no obvious separation among colonies in ordination space (Figure 2). Pairwise *F*_ST_ values were similarly low, ranging from 0.004 (EA–CE) to 0.14 (CC–BR and CC–SR) (Table 1), indicating only minor genetic differentiation.

**TABLE 1:** Pairwise *F*_ST_ estimates between each population of Australian white ibis in the Moreton Bay region of Queensland, Australia.

Site	Arthur Allan	BP Caboolture	Bribie	Centenary	Central Lakes	Clough Ct	DBay SES	Esme Ave	Fodora Place	Humpybong	Morayfield	Ningi	Redcliffe Bot Gar	Sweeney Res
BP Caboolture	0.01													
Bribie	0.01	0.05												
Centenary	0.02	0.02	0.06											
Central Lakes	0.01	0.01	0.05	0.01										
Clough Ct	0.08	0.08	0.14	0.09	0.07									
DBay SES	0.00	0.03	0.02	0.05	0.03	0.10								
Esme Ave	0.00	0.01	0.04	0.01	0.00	0.07	0.03							
Fodora Place	0.01	0.04	0.00	0.05	0.04	0.11	0.00	0.03						
Humpybong	0.01	0.01	0.05	0.01	0.01	0.07	0.03	0.01	0.04					
Morayfield	0.01	0.01	0.05	0.01	0.01	0.08	0.04	0.01	0.04	0.01				
Ningi	0.00	0.02	0.01	0.04	0.02	0.11	0.01	0.01	−0.01	0.02	0.02			
Redcliffe Bot Gar	−0.01	0.03	0.00	0.04	0.03	0.10	0.00	0.02	−0.01	0.03	0.03	0.00		
Sweeney Res	0.05	0.04	0.09	0.06	0.04	0.13	0.07	0.04	0.07	0.04	0.04	0.07	0.05	
Woodford	0.02	0.02	0.06	0.03	0.02	0.10	0.04	0.01	0.05	0.01	0.02	0.03	0.04	0.06

**FIGURE 3:**
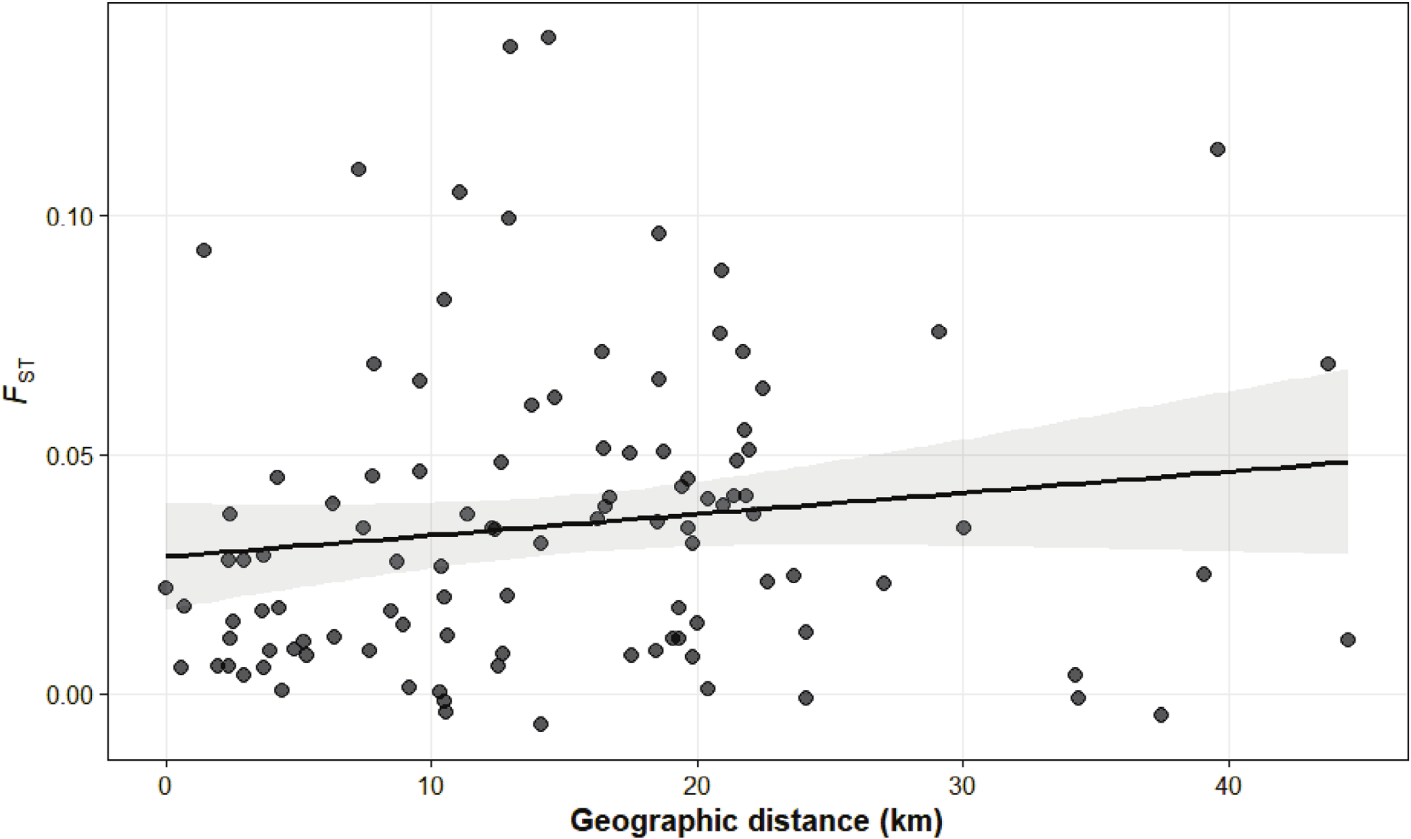
Relationship between geographic distance and linearised genetic differentiation (*F*_ST_/(1 − *F*_ST_)) among Australian white ibis colonies in the Moreton Bay region of Queensland, Australia. Statistical inference was conducted using a Mantel test (*r* = 0.155, *p* = 0.14).

Isolation‐by‐distance (IBD) analysis showed weak spatial structure. Mantel tests detected a small, non‐significant correlation between linearised *F*_ST_ and geographic distance (*r* = 0.155, *p* = 0.14; 99 permutations; Figure 3).

### 3.2 Genetic Diversity, Inbreeding, and Demographic Indicators (H2)

Across the 15 sampled colonies, genetic diversity was moderate (Table 2). Several colonies were represented by small sample sizes (*n* = 2–3), and therefore colony‐level estimates of *H*_o_, *F*_IS_, sex ratio, and N_e_ from these sites should be interpreted cautiously.

**TABLE 2:** The number of females, males and total individuals sampled along with genetic diversity (*H*_e_, *H*_o_ and *F*_IS_) estimates for each population of Australian white ibis in the Moreton Bay region of central eastern Australia.

Site	Females	Males	Total	*H*_e_	*H*_o_	*F*_IS_
Arthur Allan	1	2	3	0.174	0.145	0.049
BP Caboolture	8	5	13	0.159	0.129	0.142
Bribie	3	0	3	0.158	0.084	0.278
Centenary	4	5	9	0.161	0.140	0.099
Central Lakes	21	18	39	0.163	0.134	0.155
Clough Ct	1	2	3	0.134	0.107	0.044
DBay SES	2	1	3	0.162	0.101	0.195
Esme Ave	22	16	38	0.163	0.125	0.194
Fodora Place	2	1	3	0.162	0.088	0.256
Humpybong	19	19	38	0.164	0.140	0.124
Morayfield	8	11	19	0.163	0.134	0.139
Ningi	2	0	2	0.162	0.117	0.103
Redcliffe Bot Gar	1	2	3	0.166	0.105	0.188
Sweeney Res	1	2	3	0.152	0.144	−0.037
Woodford	2	3	5	0.155	0.126	0.123

Observed heterozygosity (*H*_o_) ranged from 0.08 (Bribie) to 0.14 (Sweeney Res), while expected heterozygosity (*H*_e_) ranged from 0.13 (Clough Ct) to 0.17 (Redcliffe Bot Gar). Inbreeding coefficients (*F*_IS_) were generally low, consistent with widespread mixing, but slightly elevated at Bribie (0.28) and Fodora Place (0.26), suggesting local constraints on gene flow possibly related to isolation (Bribie) or small colony (Fodora Place).

Kinship analysis identified six close‐kin pairs, including two parent–offspring pairs (one within‐colony, one between colonies) and four sibling pairs, half of which were cross‐colony, providing direct evidence of recent dispersal among colonies. Effective population size (*N*_e_) estimates ranged from 142 to 405 (total *N*_e_ = 215).

SNP‐based sexing successfully assigned sex to 64 previously unsexed birds (35 F, 29 M). Overall, the dataset yielded a balanced sex ratio (1.11F:1M) with no significant regional skew (*χ* ^2^ = 9.14, *p* = 0.822; Fisher's exact *p* = 0.889; Table 2).

To test whether spatial genetic structure differed by sex, we ran sex‐stratified isolation‐by‐distance analyses. Mantel correlations were non‐significant for both sexes (males: *r* = −0.023, *p* = 0.60; females: *r* = 0.054, *p* = 0.217), indicating no detectable IBD signal in either group at this scale (Figure S1). Ordination patterns were also qualitatively similar between sexes (Figure S2). Together, these results provide no evidence of sex‐biased dispersal within the study region.

### 3.3 Landscape Influences on Connectivity and Diversity (H3)

Isolation‐by‐resistance analyses showed that neither resistance surface improved the association between genetic and spatial distances. The urban‐proximity model yielded *r* = 0.196 (*p* = 0.13), and the landscape‐variation model yielded *r* = 0.141 (*p* = 0.18). Thus, resistance distances provided no detectable explanatory gain over straight‐line distance at this spatial scale, reinforcing that the urban matrix imposes, at most, weak resistance to ibis movement.

**FIGURE 4:**
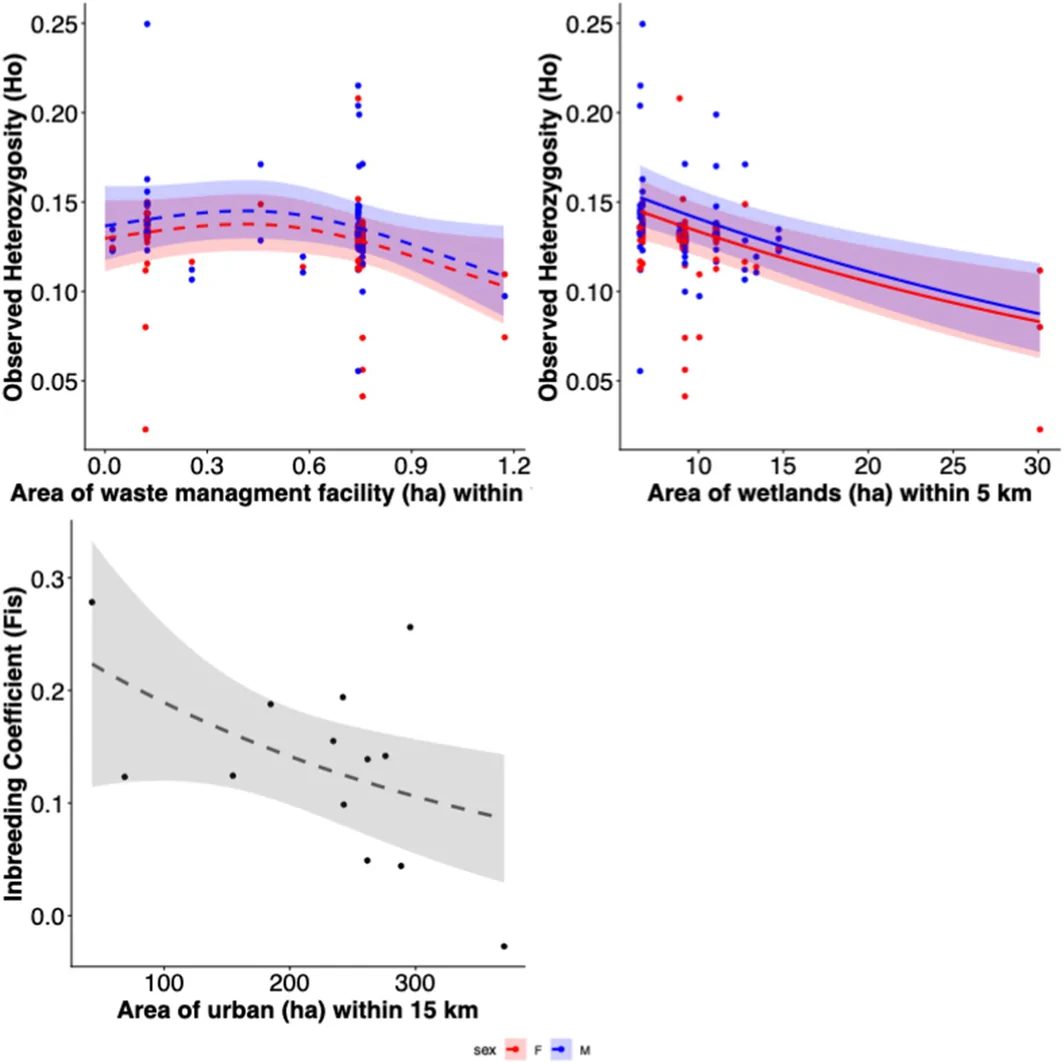
Relationships identified by the best‐fit full‐subsets generalised additive models (FssGAMMs) explaining genetic diversity and inbreeding in Australian white ibis ( *Threskiornis molucca* ) colonies in Moreton Bay, Australia. (A) Relationship between waste‐management facility area within 10 km of a colony (ha) and observed heterozygosity (*H*_o_). (B) Relationship between wetland area within 5 km of a colony (ha) and observed heterozygosity (*H*_o_). (C) Relationship between urban land cover within 15 km of a colony (ha) and inbreeding coefficient (*F*_IS_). Dashed lines indicate non‐significant (i.e., *p* > 0.05) patterns. Shaded bands represent modelled 95% confidence intervals. Male and female predictions are shown separately where applicable (males = blue, females = red).

The observed heterozygosity (*H*_o_) of individual ibis decreased significantly as the area of wetlands within 5 km (*F* = 14.956, *p* = 0.0001) increased and males were significantly higher than females (*F* = 4.9, *p* = 0.03) (Figure 4). The highest observed heterozygosity (approximately *H*_o_ 0.16 for both male and female ibis) was observed at sites with less than 10 ha of wetlands within a five‐kilometre radius.

Conversely, there was a weak negative trend between waste‐facility area within 10 km and observed heterozygosity, but this relationship was not statistically significant (*F* = 2.004, *p* = 0.105). *F*_IS_ decreased marginally with urban land cover (*F* = 3.622, *p* = 0.083), and these relationships were consistent for males and females (Figure 4).

## 4 Discussion

We used genome‐wide SNP data to test how urbanisation shapes population structure and processes in a highly adaptable native species, the Australian white ibis. Consistent with our predictions, we found very weak population structure, minimal genetic differentiation among colonies and no signal of isolation by distance, indicating high connectivity across the landscape (H1). Genetic diversity was moderate overall, with low levels of inbreeding and the presence of cross‐colony kin pairs, all confirming contemporary dispersal between colonies. Furthermore, sex ratios were balanced and we found no genetic evidence of sex‐biased dispersal (H2). Finally, landscape resistance models explained little additional variance beyond geographic distance, supporting the hypothesis that urban features impose minimal barriers to movement. However, we did detect an unexpected negative relationship between wetland extent and heterozygosity, suggesting that not all habitat types are equivalent in their effects on genetic diversity (H3). Together, these results suggest that ibis colonies in the study region function more as components of a single highly connected regional population than as genetically distinct units within a classical metapopulation. Frequent movement and gene flow appear sufficient to maintain connectivity across the study area despite differences among colonies in habitat composition and local demographic characteristics.

### 4.1 Population Structure

Connectivity between colonies is fundamental to population persistence, allowing for dispersal, migration, gene flow, and habitat establishment or recolonisation (Zeller et al. 2020). Population connectivity can be inferred by the extent to which genetic information is shared between colonies and individuals (De Young and Honeycutt 2005). Here, we found evidence of high levels of connectivity, little evidence of significant genetic partitioning, and kinship across colonies in the sites sampled, demonstrating congruousness in the population. High connectedness in the PCA between individuals from all colonies indicates that they were not genetically isolated from one another; and that individuals may have genetic information from other conspecific ibis populations in the broader region (Alvarez 2008). Additionally, low *F*_IS_ values in some colonies suggest outbreeding with genetically distinct populations outside the sampled region (Edmands 2007). Our findings are consistent with previous ibis research across the east coast of Australia. For example, colonies from seemingly geographically isolated populations, including those from both coastal and inland Queensland, New South Wales, and Victoria, showed moderate heterozygosity and evidence of connectivity (Davis et al. 2021). It is likely that this connectivity is associated with ibis' well‐known behavioural plasticity and mobility (N. J. Murray and Shaw 2009). The lack of structure suggests that urban landscapes, rather than fragmenting ibis populations, may actually facilitate connectivity by providing abundant, predictable resources that promote movement among sites (J. Martin et al. 2012; J. M. Martin et al. 2011; Willis et al. 2024). Indeed, ordination analyses revealed strong genetic overlaps between colonies and minimal variance explained by principal components (1.28%, 1.14%), characteristic of pan‐mictic populations.

The lack of population structure suggests that urban landscapes, rather than fragmenting ibis populations, may facilitate connectivity by providing abundant, predictable resources that promote movement among sites. This supports the interpretation that for this species, the urban environment may be facilitating or potentially forcing rather than constraining movement, dispersal and overall genetic connectivity. While many studies have found that urban landscapes act as barriers to gene flow (Delaney 2014; Schell 2018), there is some evidence that urban‐adapted species such as rock doves ( *Columba livia* ) and Javan mynas ( *Acridotheres javanicus* ) remain highly dispersive and connected within the urban matrix (Carlen and Munshi‐South 2021; Low et al. 2018). Despite the broader global trend of increased genetic structuring in urban bird populations, ibis appear unusually well‐connected across the urban landscape. Whether this is a direct result of morphology, behavioural plasticity or other factors unique to this species is unknown.

### 4.2 Demographic Processes and Dispersal

Although we can only make limited conclusions regarding dispersal and demographic processes based on genetic data, our results provide multiple lines of evidence that ibis colonies in the study region form a single, interconnected population rather than a classic metapopulation (Wells and Richmond 1995). Moderate heterozygosity across colonies, and generally low *F*_IS_ estimates are consistent with a large, stable population with ongoing gene flow via frequent movement between colonies, preventing inbreeding (Bicknell et al. 2012; Leighton and Echeverri 2016; Beccardi et al. 2024; Saatoglu et al. 2025). Only two colonies, Bribie Island and Fodora Place, exhibited elevated inbreeding coefficients, suggesting potential local constraints on gene flow. Bribie Island's distinctiveness is perhaps unsurprising: as an island connected by a bridge but separated from the mainland by large expanses of non‐urban landscape, it may experience reduced dispersal relative to other sites (discussed above). The elevated inbreeding at Fodora Place is more difficult to interpret but may reflect small local colony size or sampling artefact. These outliers demonstrate that even within a highly connected metapopulation, local factors can occasionally limit dispersal and promote mild inbreeding, although such effects do not appear to impact the broader population. One colony, Sweeney Reserve (SR), exhibited a slightly negative *F*_IS_ value, indicating a modest excess of heterozygotes relative to Hardy–Weinberg expectations. Interpretation should be cautious given the small sample size from this colony. Estimates of *H*_o_, *F*_IS_, sex ratio, and N_e_ are expected to be less precise when based on only two or three individuals and should therefore be viewed as exploratory indicators rather than robust colony‐level demographic estimates. Importantly, conclusions regarding regional connectivity and genetic structure were supported consistently across analyses incorporating the full dataset.

The detection of cross‐colony kin pairs (i.e., parents‐offspring and sibling pairs) provides another line of direct evidence of recent dispersal and gene flow among colonies. This suggests that individuals are mating between colonies with multiple partners in one breeding season, and that birds travel between sites in Moreton Bay, like previously reported in studies of ibis in the Sydney area (D. Corben 2008; Davis et al. 2021; J. M. Martin et al. 2011; A. C. M. Smith and Munro 2011; Thomas et al. 2014). For example, both non‐urban and urban ibis colonies disperse regularly during the breeding season throughout Australia (D. Corben 2008; Davis et al. 2021; Thomas et al. 2014).

Oftentimes, sex contributes to avian dispersal, whereby dispersal is unbalanced between sexes (Li and Kokko 2019; Zsinka et al. 2025). Our results, however, indicate that sex‐biased dispersal is unlikely, since a balanced sex ratio with no strong indication of regional skews suggests both sexes contribute equally to population dynamics (Li and Kokko 2019). This is further supported by our sex‐stratified analyses, which showed that isolation‐by‐distance signals were absent in both males and females, and ordinations were qualitatively similar. One trend that could be investigated further in the future is that males did consistently show marginally higher observed heterozygosity than females across colonies. However, our sample sizes do preclude broad interpretations of this pattern.

### 4.3 Landscape Influences on Connectivity and Diversity

The role of landscape characteristics on genetic connectivity of colonies is a priority topic for many researchers, because it can give insight into management considerations or increase our understanding of the mobility of organisms especially across human‐altered habitats. Here, we found limited evidence for the influence of landscape composition on patterns of ibis genetic diversity across our highly urbanised study region. Consistent with H3, we found limited evidence that landscape composition influenced genetic differentiation among colonies. However, landscape composition did appear to influence within‐colony genetic diversity, particularly through the relationship between wetland extent and heterozygosity. Our IBR tests indicated that resistance distances derived from the colony network and background landscape did not explain genetic differentiation any better than geographic distance alone. Together with the non‐significant IBD signal, these results suggest that, at the regional scale considered here, urban features impose weak resistance to movement for ibis, a pattern consistent with a highly mobile species routinely traversing the urban matrix. A limitation of the isolation‐by‐resistance analysis is that the resistance surfaces used were only indirectly related to known drivers of ibis movement. The surfaces were intended as exploratory representations of spatial variation and were not parameterised using habitat characteristics known to influence ibis ecology, such as wetland availability, landfill proximity, or urban land cover. Consequently, the lack of support for resistance effects should be interpreted cautiously. Future studies incorporating biologically informed resistance surfaces based on habitat use and movement data may provide stronger insight into the landscape features influencing ibis connectivity. Furthermore, because not all colonies within the region were sampled, some sources of genetic variation may remain untested. Nevertheless, the selected colonies encompass a broad range of landscape contexts representative of the urbanised Moreton Bay region.

Observed heterozygosity decreased significantly as wetland area increased, suggesting that ibis colonies occupying landscapes with abundant wetlands may be more likely to breed locally, reducing the genetic mixing between breeding colonies (D. Corben 2008; Davis et al. 2021). Conversely, individuals in regions with less wetland may frequently move between sites to access resources, increasing opportunities for outbreeding and gene flow (Claramunt et al. 2011; Swift et al. 2021). As waste management facilities and urban land cover did not significantly affect observed heterozygosity or inbreeding, it indicates that urbanised environments do not strongly restrict genetic connectivity, a trend found within other urban‐commensal species (Carlen and Munshi‐South 2021; Combs et al. 2018). Despite the relatively low variation in habitat composition across the Moreton Bay region, ibis populations can maintain genetic diversity, reinforcing their ability to persist within urban environments (J. Martin et al. 2010; Willis et al. 2024).

### 4.4 Implications for Management and Conservation, and Future Directions

Given the connectivity between ibis colonies throughout the region, management should consider incorporating large‐scale and coordinated practices, whilst being careful not to impact inland colonies. High connectivity across the region means that ibis colonies are likely resilient to localised disturbances, but also that management interventions at one site may have far‐reaching effects across the metapopulation. Previous research in the region details that ibis and their nests are most abundant in habitats within 10 km of a waste management facility (Willis et al. 2024). Further, nests in the region were most abundant on water‐bound islands (such as in parklands), with a tree density of greater than 0.5 trees/m^2^ (Willis et al. 2024). If wetlands truly support distinct population processes (lower diversity, potentially more stable demography), they may warrant special conservation attention, especially inland or away from urban centres. This could help reduce potential conflict with local human populations, especially if paired with restricting access to key food resources (such as waste management facilities). More generally, this study demonstrates that even species considered ‘urban adapters’ can exhibit complex, context‐dependent responses to landscape heterogeneity, reinforcing the value of integrating genomic data with spatial and demographic information in wildlife management. Because our sampling focused on colonies within a single urbanised region, we cannot directly evaluate genetic differences between urban and non‐urban ibis colonies. Future work incorporating inland and less urbanised colonies would provide a stronger test of how urbanisation influences population genetic structure across the species' range.

This study demonstrates that Australian white ibis in the Moreton Bay region function as a highly connected regional population, with weak genetic structure, moderate diversity, low inbreeding, and minimal landscape resistance. Furthermore, in contrast to other studies, urbanisation appears to facilitate connectivity, rather than fragmenting ibis populations, by creating a resource‐rich matrix that promotes movement and gene flow. Variability between sites or colonies, and the relationship between wetlands and genetic diversity, however, show that even highly connected species can exhibit context‐dependent responses to landscape heterogeneity. As urbanisation continues to reshape eastern Australian coastlines, understanding the genetic architecture of connectivity in adaptable native species will be critical for predicting population trajectories and designing effective management strategies. The ibis, despite its polarising presence, can serve as a model system for understanding how wildlife navigates the Anthropocene.

## Author Contributions

**Dominique A. Potvin:** conceptualization (lead), data curation (lead), formal analysis (equal), funding acquisition (equal), investigation (equal), methodology (lead), project administration (lead), resources (lead), supervision (lead), visualization (supporting), writing – original draft (equal), writing – review and editing (equal). **Angela G. Webb:** data curation (equal), formal analysis (equal), investigation (equal), writing – original draft (equal). **Tatiana Wendy Fonseca:** investigation (equal), methodology (equal), writing – original draft (equal). **Jessica Gorring:** conceptualization (equal), funding acquisition (equal), investigation (equal), project administration (equal), writing – review and editing (equal). **Caitlin S. Willis:** investigation (equal), methodology (equal), visualization (equal), writing – original draft (equal), writing – review and editing (equal). **Erin K. Wills:** investigation (equal), methodology (equal), visualization (equal), writing – original draft (equal), writing – review and editing (equal). **Ben L. Gilby:** conceptualization (equal), formal analysis (equal), investigation (equal), methodology (equal), project administration (equal), visualization (equal), writing – original draft (equal), writing – review and editing (equal).

## Funding

This work was supported by City of Moreton Bay.

## Disclosure

Benefit‐Sharing Statement: Benefits from this research accrue from the sharing of our data and results on public databases as described above.

## Conflicts of Interest

The authors declare no conflicts of interest.

## Supporting information


**Figure S1:** Isolation‐by‐distance analysis among Australian white ibis colonies in Moreton Bay, Australia. Each point represents a pairwise comparison between two colonies (*n* = 105 comparisons). Genetic distance was measured as pairwise FST estimated from SNP markers, and geographic distance was calculated as the great‐circle distance (km) between colony locations. Lines shows the fitted least‐squares linear regression used to visualise the relationship between genetic and geographic distance.
**Figure S2:** Principal Coordinates Analysis (PCoA) of Australian white ibis genotypes, displayed separately for females and males. Points represent individual birds and are coloured according to colony of origin.

## Data Availability

All data and code used in the manuscript can be accessed at https://doi.org/10.6084/m9.figshare.31650106.
