# Supplementary material for: High Genetic Connectivity and Weak Structure Shape Australian White Ibis Metapopulation Dynamics Across an Urban Coastal Landscape
Source: Ecol Evol. 2026 Aug 28;16(9):e74247. doi: 10.1002/ece3.74247 (PMC13525338; doi:10.1002/ece3.74247)
Supplement: Supplementary file 1 [file ECE3-16-e74247-s001.docx]

Supplementary Materials for Ibis Genetics manuscript

Figure SF1. Isolation-by-distance analysis among Australian white ibis colonies in Moreton Bay, Australia. Each point represents a pairwise comparison between two colonies (n = 105 comparisons). Genetic distance was measured as pairwise FST estimated from SNP markers, and geographic distance was calculated as the great-circle distance (km) between colony locations. Lines shows the fitted least-squares linear regression used to visualise the relationship between genetic and geographic distance.

Figure SF2. Principal Coordinates Analysis (PCoA) of Australian white ibis genotypes, displayed separately for females and males. Points represent individual birds and are coloured according to colony of origin.
